# Beyond top-down: community co-creation approaches for sustainable dengue vector control

**DOI:** 10.1080/16549716.2024.2426348

**Published:** 2024-11-08

**Authors:** Peter Dambach, Valérie R. Louis, Claire J. Standley, Carlos Alberto Montenegro-Quiñonez

**Affiliations:** a Heidelberg Institute of Global Health, University of Heidelberg, Heidelberg, Germany; b Center for Global Health Science and Security, Georgetown University, Washington, DC, USA; c Instituto de Investigaciones, Centro Universitario de Zacapa, Universidad de San Carlos de Guatemala, Guatemala City, Guatemala

**Keywords:** Dengue fever, co-creation, community engagement, vector control, participatory approaches, participatory map creation, citizen science

## Abstract

Dengue fever, a mosquito-borne viral illness transmitted by *Aedes* mosquitoes, continues to be a significant public health burden in tropical and subtropical regions. Traditional vector control methods, primarily reliant on insecticides and larvicides, face challenges because of emerging insecticide resistance and limited community engagement. This narrative review explores co-creation as a collaborative approach to dengue control, where communities actively participate in designing and implementing solutions. Through an examination of existing literature, we discuss the rationale for co-creation, the various methods employed, evidence for effectiveness, challenges, and other items. Findings from previous studies suggest that co-creation can empower communities by fostering a sense of ownership and responsibility for dengue control efforts. Using local knowledge and insights, co-creation approaches have also been shown to identify and address specific community needs and preferences, leading to more contextually relevant interventions. Additionally, co-creation initiatives have demonstrated success in promoting behavior change within communities, leading to increased uptakes of preventive measures such as proper waste management and use of personal protective measures. However, challenges such as building trust and collaboration, addressing power dynamics, and ensuring long-term sustainability remain critical factors that are essential to foster collaboration, empower communities, and develop sustainable strategies for dengue control in affected regions.

## Paper context

- **Main findings:** Traditional top-down approaches often fail to achieve meaningful community engagement, limiting their overall impact. However, community co-creation methods, which emphasize collaboration between researchers and community members, have shown promise in other public health initiatives but remain underutilized in dengue control efforts.
- **Added knowledge:** This review presents different available community co-creation approaches, in what way they go beyond simple community participation and how this contributes to increased acceptability and sustainability of dengue control projects.
- **Global health impact for policy and action:** This study provides arguments for integrating community co-creation methods into dengue vector control programs, potentially transforming standard practices.

## Background

Dengue fever, caused by a mosquito-borne flavivirus, continues to be a major public health concern globally. The World Health Organization (WHO) reported around 5 million dengue cases for 2023 [1] with some models hinting that this represents substantial underestimates of the true number [2]. Dengue poses a significant economic burden, with estimates exceeding $8–9 billion annually in direct healthcare costs [3]. The primary vectors for dengue transmission are *Aedes aegypti* and *Aedes albopictus* mosquitoes that thrive predominantly in urban and peri-urban environments and exhibit different behaviors to those of malaria vectors with a strong anthropophilic behavior, adding complexity to vector control efforts for dengue and forcing control initiatives to diverge from well-established malaria management approaches [4,5]. Currently, dengue vector control methods rely heavily on insecticides to target adult mosquito populations and breeding site removal and larvicides to control larval populations. The emergence of insecticide resistance in *Aedes* mosquitos has significantly compromised the effectiveness of these methods [6–8] and requires the use of advanced insecticides or the increased use of alternative methods. Additionally, these approaches are often designed and applied in a top-down manner, leading to a lack of community engagement and acceptance which can compromise long-term program success and reduce cost-effectiveness [9].

In fact, a wide body of evidence shows that community participation can overcome some of the disadvantages of top-down approaches and contribute to the success of mosquito control interventions [10–14]. Community participation involves members of a community engaging in activities or projects. The level of participation can range from simple information to citizen control and the definition of community participation is likely to slightly vary between authors [15]. A widely employed interpretation of community participation sees community members typically play a supportive or consultative role, providing input, feedback, or labor. They contribute by offering insights, voicing concerns, and assisting in the implementation of plans, but they usually do not have control over the decision-making process. Projects and initiatives in community participation are generally designed and driven by external entities, with the community participating within an existing framework rather than creating it. In this context, a community can be defined as the people living in one particular area or people who are considered as a unit because of their common interests, social group, or nationality. The level of engagement can range from low to moderate, often involving attending meetings, filling out surveys, or volunteering for specific tasks. Sometimes participatory interventions may produce results that were never envisaged at the outset [16]. Although community participation does address and fix some of the typical shortcomings from top-down approaches often making them more successful, in many instances the projects do not involve communities enough to make them fully identify with interventions, take ownership and guarantee long-term sustainability [10,17,18].

Community co-creation goes a step further, being a collaborative process where community members and external entities work together from the inception to the completion of a project, emphasizing joint ownership and shared decision-making [19]. In co-creation, community members are equal partners who actively contribute to every stage, including planning, decision-making, implementation, and evaluation [20]. Projects often emerge from the community’s needs and ideas, with external entities acting as facilitators or partners who provide resources and expertise while respecting the community’s leadership and vision. Engagement in community co-creation is high, with community members deeply involved in all aspects of the project, from brainstorming and designing to managing [19,21]. As a result, community members have substantial influence over the outcomes, with decisions being made collaboratively to ensure that the project aligns closely with the community’s goals and values [22]. Examples for successful implementation range from the co-creation of a dengue early warning system for the health sector in Barbados [23] to the more general co-creation and dissemination of health-related knowledge [19]. The impact of co-creation in community-based health systems goes even further and, in a systematic review, was found to achieve impact at the level of research [21].

In this narrative review, we aim to explore the potential of co-creation approaches that go beyond simple community participation for effective and sustainable dengue control. We discuss the rationale for co-creation, various methods employed, evidence for effectiveness, challenges, and considerations. By examining these approaches, we seek to provide insights into how co-creation can empower communities and transform dengue control efforts.

## Co-creation methods in dengue control

### Rationale

Co-creation provides a collaborative approach where communities actively participate in the design and implementation of dengue control initiatives; it encompasses co-design and co-production of interventions [20,24]. It empowers communities by granting them agency and responsibility for their health and environment, thereby increasing their motivation to actively participate in control activities. This sense of ownership is pivotal for ensuring the sustainability of interventions beyond the initial program period [25–27], and in this way, aligns with the conceptual approach taken by the social-ecological systems theory [28]. Communities possess invaluable insights into their local environment, including the identification of potential mosquito breeding sites, understanding mosquito behavior patterns, and awareness of cultural practices that influence disease transmission [19,28]. Integrating this knowledge into scientifically backed control strategies enhances their effectiveness and context-specificity. Involving communities in the design of interventions instills a sense of responsibility and ownership, fostering greater motivation to adopt preventive behaviors. This includes such practices as waste management, modifications to water containers, and the correct use of personal protective measures [29,30]. Effective co-creation cultivates trust and collaboration between communities and public health officials. This encourages open communication channels, enabling the early identification of issues and challenges, and facilitating a more responsive and effective control program.

### Community mobilization and capacity building

The foundation for co-creation efforts can be laid through interactive community workshops and awareness campaigns. Workshops can utilize various participatory techniques like group discussions, role-playing activities, and presentations delivered by public health officials and community leaders. Awareness campaigns can use multiple channels such as community radio broadcasts, distribution of educational pamphlets, public schools, and street theatre performances to raise awareness about dengue transmission, prevention measures, and the importance of community participation. These activities equip community members with the knowledge necessary to actively participate in co-creation initiatives [31]. However, co-creation goes beyond simply raising awareness. Capacity-building workshops can be conducted to equip community members with practical skills relevant to dengue control and vectors of other diseases [32]. This may involve training on larval source identification and management, proper use of household insecticides, and strategies for promoting behavior change within social networks. Investing in capacity building empowers communities to take ownership of control efforts and ensures the sustainability of interventions beyond the initial program period [33,34].

### Participatory map creation

Participatory map creation involves community members actively collaborating with public health officials and researchers to identify and map potential mosquito breeding sites within their neighborhoods. This method leverages local knowledge and perspectives that might be missed by traditional top-down approaches. The mapping process can be facilitated using various tools, including paper maps overlaid with transparent sheets, digital mapping apps on mobile devices, or even creating three-dimensional models using locally available materials [35]. By co-creating these maps, communities not only identify breeding sites but also prioritize areas for intervention based on factors like proximity to households and difficulty of access [36].

### Community-based participatory research

Community-Based Participatory Research (CBPR) is a collaborative approach to research that tackles social and health disparities. While the term co-creation encompasses a wider range of collaborative efforts and is aimed at innovation and solution development, CBPR is specifically rooted in the research process. It emphasizes social justice by actively involving community members, alongside researchers and organizational representatives, in all stages of the research process [22,37]. This means community members contribute their expertise and lived experiences, leading to a deeper understanding of the issue and the development of solutions that directly benefit their community [21].

### Participatory action research

Participatory Action Research (PAR) is a collaborative research approach that goes a step further than community-based participatory research; here researchers and community members work together as equal partners throughout the research process. Compared to CBPR PAR places a stronger emphasis on action and the iterative process of implementing changes based on research findings, while CBPR focuses more on research partnership and generating relevant knowledge. In the context of dengue control, participatory action research can be a powerful tool for co-creation. Community members actively participate in all stages of the research, from identifying local priorities and research questions to designing and implementing interventions, collecting data, and analyzing results. This collaborative approach ensures that the research is not only scientifically sound but also addresses the specific needs and challenges faced by the community [38]. Furthermore, PAR empowers communities to become active agents of change, fostering a sense of ownership over the research findings and their application in developing effective control strategies.

### Citizen science initiatives

Citizen science initiatives harness the power of volunteers from the community to collect valuable data on mosquito populations and breeding sites. To date, they have proven their worth particularly in the global north while data from the global south is more scarce [39,40]. Training programs can be conducted to equip volunteers with the skills necessary to identify adult *Aedes* mosquitos and their larvae, as well as monitor potential breeding sites [41]. Citizen science projects often utilize smartphone applications to facilitate data collection and reporting. Volunteers can use these apps to record the location and type of breeding sites they encounter, along with additional observations such as mosquito abundance. The collected data can then be uploaded to a central database accessible to public health officials and researchers. This citizen-generated data is valuable for informing targeted vector control efforts, identifying areas with high mosquito populations, and evaluating the effectiveness of control interventions [42,43]. Citizen science not only contributes valuable data but also fosters a sense of community ownership and empowers residents to play an active role in dengue prevention by monitoring their own neighborhoods.

## Co-creation of vector control interventions

**Figure 1.**
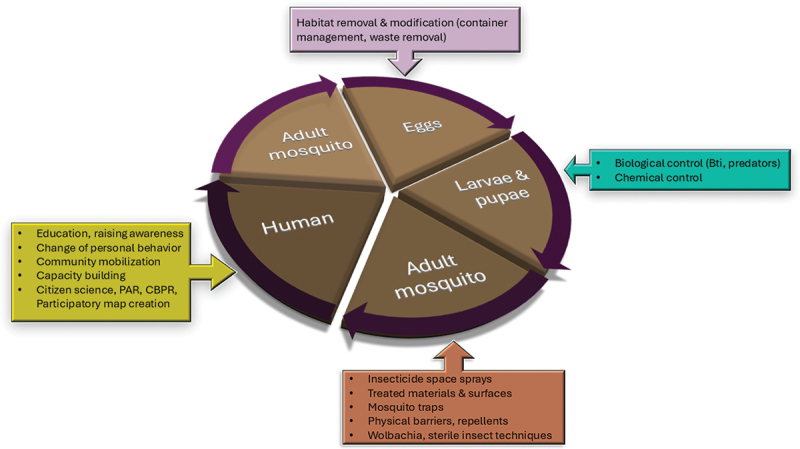
Systematic framework illustrating where community-based interventions can be applied on the epidemiological pathway from mosquito oviposition to humans.

Co-creation methods in dengue control extend beyond simply raising awareness and mobilizing communities. Empowered communities play a vital role in shaping and implementing specific interventions, whose efficacy has ideally been proven in randomized controlled trials, tailored to their local context [44–46]. But even before entering the stage of interventions, co-creation can go as far as being employed in the design of early warning systems [23]. In the following, we present how co-creation can be applied to various dengue control strategies along the epidemiological pathway from mosquito oviposition to human health (Figure 1). These strategies encompass a range of activities, including habitat management to reduce mosquito breeding sites, the introduction of biological control agents, community-driven surveillance programs, and education campaigns tailored to specific behavioral practices. By engaging communities at each step, these co-created strategies lead to a more holistic and sustainable approach to dengue control that is resilient to the unique challenges of each locale.

### Habitat removal and modification

An important strategy to control a variety of such vector-borne diseases as dengue, malaria and others is the removal or alteration of their breeding grounds. Participatory map creation exercises can help to identify potential mosquito breeding sites within the community. Residents with their in-depth knowledge of the local environment can pinpoint neglected areas, discarded items, and hidden water storage containers that might serve as breeding grounds. Through facilitated discussions, the community can then prioritize areas for intervention based on factors like proximity to households, difficulty of access, and potential impact. Following the identification of breeding sites, co-creation facilitates community ownership by organizing clean-up campaigns. Residents actively participate in removing unnecessary items like old tires, plastic bottles, and other potential containers that can hold stagnant water [47].

### Physical barriers

Community workshops can be used to educate residents on the importance of window screens, door netting and closing eaves for preventing adult mosquito entry [48,49]. Co-creation can involve exploring locally available and affordable materials for screen construction or repair as has been tested against malaria [50]. Furthermore, community mobilization can facilitate bulk purchases of screening materials at a discounted price, making them more accessible to residents

### Biological control

Exploring the use of mosquito predators like copepods, turtles, guppies, and others can be a sustainable and environmentally friendly approach that has been used successfully when supported and run by communities [11,41]. Equally, the release of biological toxins, such as *Bacillus thuringiensis israelensis* (*Bti*) and *Bacillus sphaericus* (*Bs*) has heavily relied on community participation [51,52]. Co-creation workshops can go a step further and introduce residents to the concept of biological control and involve them in discussions about the feasibility and local availability of these predators. Additionally, communities can be empowered to manage breeding sites suitable for the chosen predators, ensuring their long-term survival and effectiveness. Additionally, the co-creation approach can involve brainstorming solutions for managing water storage containers, such as covering them securely or introducing predators like fish, biological larvicides, or bacteria toxins (*Bacillus thuringiensis*).

### Trapping and elimination

Deploying adult mosquito traps can be a valuable tool in monitoring mosquito populations and assessing the effectiveness of control efforts. Co-creation can involve training community members on trap placement, maintenance, and proper disposal of collected mosquitoes but can even go as far as locally manufacturing traps and other utensils [53]. This fosters a sense of ownership and empowers residents to actively participate in mosquito surveillance. Ovitraps, designed to attract and capture egg-laying female mosquitoes, can be another tool for targeted control [54]. Co-creation workshops can educate residents on the purpose and use of ovitraps, and they can be involved in building and strategically placing them in areas identified during participatory map creation exercises. Community members can then be trained to monitor ovitraps and report data on collected eggs, providing valuable insights into mosquito activity.

### Larviciding and insecticide use

It is now widely recognized that communities and key stakeholders need to be informed of and engaged in spray interventions against vector mosquitoes and such involvement has been implemented [55]. However, great potential still lies untapped when it comes to local adaptation and acceptance of those activities. Co-creation workshops could harness this, educating residents on different larvicide options, application techniques, and potential environmental risks. Additionally, the community can participate in developing guidelines for safe larvicide use within their environment. Insecticide spraying against adult vector mosquitoes, such as Indoor Residual Spraying (IRS) and Indoor Space Spraying (ISS) can be a component of dengue control programs, but concerns exist about resistance development and environmental impact. Apart from entomological parameters, community acceptance and support play a major role in the success of such programs [14]. Co-creation can go beyond and facilitate discussions about how to manage and finance such interventions, their responsible use, including applying proper techniques, targeting specific areas, and minimizing exposure to non-target organisms.

### Container management

Co-creation workshops can involve brainstorming solutions for designing and strategically managing essential water storage containers. Residents can share their experiences and preferences for container covers and explain advantages and disadvantages of certain designs [56]. The community can then collaborate with local manufacturers or artisans to design and produce affordable and culturally appropriate container covers that fit their specific needs.

### Treated fabrics and repellents

Insecticide-treated clothing and bed nets can offer protection from mosquito bites. Co-creation workshops can explore the feasibility of using locally available fabrics and exploring safe, long-lasting treatment methods. Community buy-in can be fostered by involving residents in the selection of clothing styles and colors that are culturally appropriate and comfortable to wear. Furthermore, traditional, and locally available repellants such as neem oil have shown efficacy [57,58] and can be analyzed for availability and locally produced and marketed [59,60].

### Wolbachia, microsporidia and sterile insect techniques

Community co-creation can also be effectively applied to more technical vector control strategies involving bacteria, fungi, or sterile insect techniques. However, given the complexity of these methods, co-creation is more likely to play a crucial role in innovating and enhancing the distribution and release processes, as well as in engaging and gaining the support of the community as has been shown is several field trials [61,62].

## Challenges and considerations for co-creation

Community co-creation of interventions in dengue vector control can face several drawbacks. The process can be expensive and time-consuming, often requiring substantial resources for engagement, training, and ongoing support. Additionally, involving the community may create high expectations that cannot be fully met, leading to potential disillusionment. Political will is also crucial; without strong commitment from local leaders and policymakers, the efforts may lack the necessary support for sustainable implementation and scaling. Recent research points out that in many of the currently field-tested approaches referred to as co creation the communities were rarely involved in the entire cycle of decision-making, which may limit the social change and acceptability that was intended by the action [63] and that despite their success on different levels, the impact on actual health outcomes needs further and more robust research [46,64]. While co-creation offers a promising approach for dengue vector control, implementing these methods effectively requires acknowledging and addressing potential challenges [65]. In the following, we review some important key factors that need to be met to ensure the long-term sustainability of co-creation initiatives. The following five key factors are of immense importance for the successful implementation of co-creation approaches. The first three key factors are issues that pose particular challenges when working with and involving communities, while the last two are important elements in the conduct of every project.

### Building trust and collaboration

Effective co-creation requires trust and open communication between public health officials, researchers, and community members. Addressing historical mistrust, power differentials, and cultural barriers is essential for meaningful engagement. Prioritizing community engagement from the outset, actively listening to local concerns, and ensuring equitable participation in decision-making processes are crucial for building trust. Furthermore, fostering collaboration among different stakeholders within the community, such as local leaders, faith-based organizations, and youth groups, can broaden ownership and strengthen the program’s effectiveness [37].

### Addressing power dynamics

Co-creation efforts must be mindful of existing power dynamics within communities, which may marginalize certain groups or limit their participation [66]. Ensuring inclusivity and equitable representation of diverse voices is critical for co-creation to be effective and sustainable. In many societies around the world, customs dictate which individuals have the right to express their opinions and participate in the voting process. This must also be considered when carrying out co-creation activities. Approaches such as participatory planning, consensus-building exercises, and community-led initiatives can empower marginalized groups and ensure their perspectives are heard and valued.

### Capacity building and resource mobilization

Successful co-creation relies on building the capacity of community members to actively participate in dengue control efforts. This requires investing in continuous training programs, skill-building workshops, and knowledge-sharing platforms. Securing sustainable funding sources and mobilizing resources for continuous capacity building is essential for the long-term success of co-creation initiatives [42]. Further implementation barriers can be found in the systemic condition of the implementation setting and the socioeconomic status of community members [67]. Exploring innovative financing mechanisms, public–private partnerships, and community fundraising strategies can contribute to financial sustainability and resource mobilization.

### Monitoring and evaluation

Regular monitoring and evaluation are essential for assessing the effectiveness of co-creation interventions and identifying areas for improvement. This includes tracking changes in mosquito populations, dengue incidence rates, community knowledge and behavior, and program reach [68]. Engaging community members in the monitoring and evaluation process ensures that it remains relevant and responsive to their needs. Developing robust monitoring and evaluation frameworks, incorporating community feedback mechanisms, and fostering a culture of continuous learning are essential for maximizing the impact of co-creation efforts.

### Adaptability and continuous learning

Successful co-creation requires flexibility and adaptability to respond to evolving community needs and environmental conditions. Public health officials and researchers must be prepared to adjust program strategies based on feedback from community members and emerging challenges. Investing in ongoing training, capacity-building initiatives, and knowledge-sharing networks can foster a culture of continuous learning and innovation [69]. By remaining responsive and adaptive, co-creation initiatives can remain relevant and effective in addressing emerging health threats and community needs.

## Future directions and recommendations

While co-creation in dengue control shows promise, further research and development efforts are needed to maximize its impact and scalability. The effectiveness of co-creation methods can vary significantly depending on cultural, socioeconomic, and geographic contexts, while much of their effectiveness depends on the effectiveness of the underlying interventions (e.g. community-based insecticide spraying can only be effective if the insecticide is effective). Future research should focus on tailoring co-creation approaches to specific communities and regions, taking into account local beliefs, practices, and infrastructural constraints. By adapting co-creation strategies to local contexts, interventions are more likely to be accepted, sustainable, and effective.

Community-based programs targeting *Aedes* control are frequently posited as sustainable. However, these assertions are typically based on evaluations conducted within the first year of program implementation [13,70]. Nonetheless, a key inquiry within participatory methodologies revolves around the duration of community engagement necessary to achieve enduring impacts [71]. Therefore, longitudinal studies are essential for assessing the long-term sustainability and impact of co-creation approaches on dengue incidence, community behavior change, and program effectiveness. These studies should track outcomes over time, including changes in vector populations, disease transmission rates, but equally community engagement levels and quality of the participation process [72]. By evaluating the long-term impact of co-creation interventions, researchers can identify successful strategies and areas for improvement.

Also, understanding the economic competitiveness and sustainability of co-created interventions compared to traditional top-down control methods is crucial. Additionally, there might be economic and organizational trade-offs between tailoring specific interventions to smaller groups of people versus implementing less adapted interventions in larger communities. Through trying to speed up and prioritize innovations with stakeholders, developments may be rendered more affordable, or at least define the market opportunity, thereby making investment, financial or otherwise, more attractive [45]. Future research should conduct cost–benefit analyses to assess the financial implications of co-creation approaches, including resource mobilization, implementation costs, and long-term maintenance expenses. By quantifying the cost-effectiveness of co-creation interventions, decision-makers can prioritize resource allocation and advocate for sustainable funding mechanisms.

After successful interventions have been identified, best practices and frameworks for scaling up successful co-creation interventions to broader contexts need to be developed. This involves creating training materials, capacity-building programs, and establishing intersectoral partnerships between public health agencies, community organizations, and research institutions. By disseminating successful co-creation models and supporting their replication in other settings, stakeholders can maximize the reach and impact of dengue control efforts. Collaboration and knowledge sharing are essential for advancing co-creation in dengue control. Future efforts should prioritize collaborative research initiatives, implementation research, interdisciplinary partnerships, and knowledge exchange platforms to facilitate learning and innovation.

Finally, some of the approaches discussed here using the example of dengue control have also been employed in other fields of research and against other vector-borne diseases such as malaria [73]. Since in many countries worldwide the health burden involves more than one disease, it would certainly be beneficial if co-creation efforts for diseases with similar transmission profiles, such as malaria, dengue, zika, chikungunya and others [39] could be bundled for increased efficiency.

## Abbreviations

*Bti*: *Bacillus thuringiensis israelensis*
CBPR: Community-Based Participatory Research
IRS: Indoor Residual Spraying
ISS: Indoor Space Spraying
NGO: Non-Governmental Organization
PAR: Participatory Action Research
WHO: World Health Organization
